# Correction: Jin et al. Effects of Zinc Source and Level on the Intestinal Immunity of Xueshan Chickens under Heat Stress. *Animals* 2023, *13*, 3025

**DOI:** 10.3390/ani13243748

**Published:** 2023-12-05

**Authors:** Jian Jin, Mengxiao Xue, Yuchen Tang, Liangliang Zhang, Ping Hu, Yun Hu, Demin Cai, Xugang Luo, Ming-an Sun

**Affiliations:** 1 Institute of Comparative Medicine, College of Veterinary Medicine, Yangzhou University, Yangzhou 225009, China; dz120210008@yzu.edu.cn (J.J.); mx120231060@stu.yzu.edu.cn (Y.T.); mz120201482@stu.yzu.edu.cn (L.Z.); 2 College of Animal Science and Technology, Yangzhou University, Yangzhou 225009, China; mz120211421@stu.yzu.edu.cn (M.X.); pinghu@yzu.edu.cn (P.H.); huyun@yzu.edu.cn (Y.H.); demincai@yzu.edu.cn (D.C.); 3 Joint International Research Laboratory of Important Animal Infectious Diseases and Zoonoses of Jiangsu Higher Education Institutions, Yangzhou University, Yangzhou 225009, China; 4 Jiangsu Co-Innovation Center for Prevention and Control of Important Animal Infectious Diseases and Zoonosis, Yangzhou University, Yangzhou 225009, China; 5 Joint International Research Laboratory of Agriculture and Agri-Product Safety of Ministry of Education of China, Yangzhou University, Yangzhou 225009, China

## 1. Text Correction

There were some errors in the original publication [1]. Abstract: “. ZnS and Zn-Prot M effectively increased the villus height and villus width in the jejunum and ileum at 74 and 88 days old, with the 60 and 90 mg/kg groups outperforming other groups, and” should be replaced with “, thus”.

Section 2.5: “the duodenum, jejunum, and ileum were isolated” should be replaced with “the duodenum samples were isolated”.

Section 3.2: “For the jejunum and ileum, …, there was still a tendency to improve the morphology of the promoting small intestine” should be replaced with “Therefore, Zn-Prot M remarkably outperforms ZnS in promoting the morphology of duodenum”; “Tables 2–4” should be changed to “Table 2”.

Section 3.3: “Tables 5–7” should be replaced with “Tables 3–5”.

## 2. Table Correction

The original Tables 3 and 4 should be removed. The numbering of the original Tables 5–7 should be updated as Tables 3–5, respectively.

The authors apologize for any inconvenience caused and state that the scientific conclusions are unaffected. This correction was approved by the Academic Editor. The original publication has also been updated.
