# Supplementary material for: Effects of Zinc Source and Level on the Intestinal Immunity of Xueshan Chickens under Heat Stress
Source: Animals (Basel). 2023 Sep 26;13(19):3025. doi: 10.3390/ani13193025 (PMC10571984; doi:10.3390/ani13193025)
Supplement: Supplementary file 1 [file animals-13-03025-s001.zip › animals-2614046-supplementary.pdf]

Table S1. Treatment settings for heat stress test in 61-102 days old Xueshan chickens.

**Experimental conditions**	**Dietary Zn source**	**Zn level (mg/kg)**	**Treatment group number**	**The number of individuals in each treatment group**	**The number of replicates in each treatment group**	**The number of individuals per replicate group**	**Notes**
Temperature: 9:00-17:00, 34 ± 1°C, 8 h/d, temperature is 28 ± 1°C for the remaining time periods; Humidity: 55 ± 5%	Negative control without Zn	0	Treatment Group 1	64	8	8	Zn-free basal diet
	Positive control for ZnS	50	Treatment Group 2	64	8	8	A complete diet supplemented with inorganic ZnS to meet Zn requirements in basal diet
	ZnS	30	Treatment Group 3	64	8	8	Complete diets supplemented with different zinc sources and zinc levels in basal diets
		60	Treatment Group 4	64	8	8	
		90	Treatment Group 5	64	8	8	
	Zn-Prot M	30	Treatment Group 6	64	8	8	
		60	Treatment Group 7	64	8	8	
		90	Treatment Group 8	64	8	8	

Table S2. Composition and nutritional level of the basal feed for 61-102 days old Xueshan chickens (as-fed basis).

Item	Ingredients (%)
Corn	76.27
Soybean meal	19.50
soybean oil	1.38
$CaHPO_4 \cdot 2H_2O$ [a]	0.79
$CaCO_3$ [a]	1.15
NaCl [a]	0.30
DL-Methionine [b]	0.12
L-lysine hydrochloride [c]	0.13
Micronutrients [d]	0.26
Cornstarch + Zn [e]	0.10
Total	100.00
Nutrient levels composition	
Metabolizable energy [f]	3037 (Kcal/kg)
Crude protein [g], %	15.31
Lysine [f], %	0.81
Methionine [f], %	0.36
L-Threonine [f], %	0.57
Tryptophan [f], %	0.16
Methionine+cystine [f], %	0.60
Calcium [g], %	0.69
Nonphytate P [f], %	0.22
Zn [g], mg/kg	18.33

[a] Reagent grade.

[b] Feed grade.

[c] Food grade.

[d] Provide per kilogram of diet for: Xueshan chickens aged 61-102 days – vitamin A, 6000 IU; vitamin D_3, 2250 IU; vitamin E, 16.5 IU; vitamin K_3, 1.5 mg; vitamin B_1, 1.5 mg; vitamin B_2, 4.8 mg; vitamin B_6, 2.25 mg; vitamin B_{12}, 0.015 mg; Pantothenic acid calcium, 7.5 mg; Niacin, 27 mg; Folic acid, 0.75 mg; Biotin, 0.075 mg; Choline, 750 mg; Cu ($CuSO_4 \cdot 5H_2O$), 7 mg; Fe ($FeSO_4 \cdot H_2O$), 40 mg; Mn ($MnSO_4 \cdot H_2O$), 40 mg; Se (Na_2SeO_3), 0.15 mg; I ($Ca(IO_3)_2 \cdot H_2O$), 0.50 mg.

[e] Zn supplements added in place of equivalent weights of cornstarch.

[f] Calculated values.

[g] Measured values.

Table S3. Primer sequences used in qPCR assays.

Gene	Sequence of the primer	Length of products (bp)
TNF-α	F: 5'-ATGAACCCTCCGCAGTACTC-3'<br>R: 5'-AAGAGGCCACCACACGACA-3'	200
IL-1β	F: 5'-CTCCTCCAGCCAGAAAGTGA-3'<br>R: 5'-GTAGCCCTTGATGCCCAGT-3'	109
IL-6	F: 5'-AGAAATCCCTCCTCGCCAAT-3'<br>R: 5'-AAATAGCGAACGGCCCTCA-3'	121
NFKBIA	F: 5'-CACCAACTACAACGGCCATA-3'<br>R: 5'-TGAAGGTCTACGGCCAAGTG-3'	150
MyD88	F: 5'-CAGAAAGACCTTCAGTTTGTCCA-3'<br>R: 5'-AATGACGACCACCATCCTCC-3'	165
ACTB	F: 5'-CTCCCTGATGGTCAGGTCAT-3'<br>R: 5'-ATGCCAGGGTACATTGTGGT-3'	203
